# Sex-related differences and age of peak performance in breaststroke versus freestyle swimming

**DOI:** 10.1186/2052-1847-5-29

**Published:** 2013-12-19

**Authors:** Mathias Wolfrum, Beat Knechtle, Christoph Alexander Rüst, Thomas Rosemann, Romuald Lepers

**Affiliations:** 1 Institute of General Practice and for Health Services Research, University of Zurich, Zurich, Switzerland; 2 Cardiovascular Center Cardiology, University Hospital Zürich, Zürich, Switzerland; 3 Gesundheitszentrum St. Gallen, Vadianstrasse 26, St. Gallen, 9001, Switzerland; 4 INSERM U1093, Faculty of Sport Sciences, University of Burgundy, Dijon, France

**Keywords:** Woman, Man, Endurance, Performance

## Abstract

**Background:**

Sex-related differences in performance and in age of peak performance have been reported for freestyle swimming. However, little is known about the sex-related differences in other swimming styles. The aim of the present study was to compare performance and age of peak performance for elite men and women swimmers in breaststroke versus freestyle.

**Methods:**

Race results were analyzed for swimmers at national ranked in the Swiss high score list (during 2006 through 2010) and for international swimmers who qualified for the finals of the FINA World Swimming Championships (during 2003 through 2011).

**Results:**

The sex-related difference in swimming speed was significantly greater for freestyle than for breaststroke over 50 m, 100 m, and 200 m race distances for Swiss swimmers, but not for FINA finalists. The sex-related difference for both freestyle and breaststroke swimming speeds decreased significantly with increasing swimming distance for both groups. Race distance did not affect the age of peak performance by women in breaststroke, but age of peak performance was four years older for FINA women than for Swiss women. Men achieved peak swimming performance in breaststroke at younger ages for longer race distances, and the age of peak swimming performance was six years older for FINA men than for Swiss men. In freestyle swimming, race distance did not affect the age of peak swimming performance for Swiss women, but the age of peak swimming performance decreased with increasing race distance for Swiss men and for both sexes at the FINA World Championships.

**Conclusions:**

Results of the present study indicate that (*i*) sex-related differences in swimming speed were greater for freestyle than for breaststroke for swimmers at national level, but not for swimmers at international level, and (*ii*) both female and male swimmers achieved peak swimming speeds at younger ages in breaststroke than in freestyle. Further studies are required to better understand differences between trends at national and international levels.

## Background

It was often assumed that men will outperform women in all sporting events requiring substantial physical exertion [1-3]. Tanaka and Seals [4,5] found this assumption to be true for running, cycling, and ice-skating, but found a different performance pattern in swimming. For swimming, the sex-related difference in performance was greatest in short duration events and became progressively smaller with increasing distance. Tanaka [5] estimated that women would outperform men at a swim distance of 25 km. In fact, records for the ‘English Channel Swim’ during 1900–2010 showed similar performances by women and men at a distance of 32 km [6]. Furthermore, an Australian woman holds the world record for the longest non-stop ocean swim: 196 km in 38 h 33 min [7]. The smaller body size, the higher proportion of body fat, and the shorter legs of female athletes are factors that prevent women from keeping up with men in other sports, but may contribute to their relatively high performance in swimming [8,9].

Sex-related differences in swim performance have been investigated for freestyle swimmers over distances of 50 m to 1,500 m [3,10-12], for swimmers in long-distance triathlons [13,14], and for open-water ultra-endurance swimmers [6], but no previous study examined sex-related differences for other styles of swimming. However, there is indirect evidence that the sex-related difference is smaller in breaststroke than in freestyle swimming. Active drag coefficient (C_d_), a measure of technique and performance, was lower for faster swimmers [15]. Havriluk [16] found that C_d_ was similar for men and women in freestyle swimming, but that women had a significantly lower C_d_ than men in breaststroke. Therefore, the greater power of men, which allows them to outperform women in freestyle swimming, might not provide such a great advantage in breaststroke.

The age of peak performance for a particular swimming style, rather than the age-related performance decline is of interest to athletes and coaches in order to estimate when performance starts to dwindle for the particular swimming style. With this knowledge swimmers and their coaches are able to focus on the optimal swimming style in the swimmers age group by tailoring the training program and organizing an appropriate competition schedule.

The age of peak performance seemed to have been relatively stable over time in some sports. For example, the age of Olympic gold medal winners of the men’s 100 m dash remained remarkably constant from 1896 to 1980 [3]. However, the age of peak performance can vary significantly with event and between sexes. For example, Berthelot et al. [17] found that peak performance in freestyle swimming was achieved at ~18 years of age for 1,500 m race distances, compared to ~23 years for 50 m distances. Schulz and Curnow [3] analysed performance of Olympic freestyle swimmers from 1886 to 1980, and found that women generally achieved peak performance at younger ages than men. Thus, the age of peak performance in 100 m events averaged 19.4 years for women and 21.4 years for men, while in 800 m events, peak age was 16 years for women and 20.3 years for men. However, Tanaka and Seals [12] reported that peak performance in 1,500 m freestyle swim was achieved at 30–35 years for women and 25–40 years for men, while peak performance in the 50 m freestyle swim occurred at 20–30 years for both sexes [12]. Inconsistent estimates of the age of peak performance for freestyle swimming might be partially due to the use of data from different time periods. Several studies failed to include data for swimmers younger than 19 years [1,7,8], and therefore, might have overestimated the age of peak performance.

The aims of the present study were to (*i*) compare the performance of women and men in breaststroke versus freestyle swimming using data from both athletes at national level (*i.e.* elite Swiss swimmers) and at international level (*i.e.* finalists in the FINA World Swimming Championships), and (*ii*) examine the age of peak performance for top female and male swimmers at different distances in both swimming styles. We hypothesized that (*i*) sex-related differences in performance would be smaller for breaststroke than for freestyle swimming, (*ii*) sex-related differences in performance would decline with increasing race distance, and (*iii*) the age of peak performance would be similar for breaststroke and freestyle swimming for both sexes.

## Methods

Race results were analyzed for all female and male breaststroke and freestyle swimmers at national level from the Swiss high score list, and for female and male swimmers at international level from the finals in the FINA (Fédération Internationale de Natation) World Swimming Championships. Data included 50 m, 100 m, and 200 m race distances. The data were obtained from the ‘Swiss Swimming Federation’ (http://rankings.fsn.ch/) for the years 2000 – 2010 and from the FINA (http://www.fina.org) for the years 2003 – 2011.

Due to the low number of participants per age group and a high variability in performance by Swiss swimmers during earlier years, analyses were limited to data from 2006 through 2010 for athletes at national level. The ‘Swiss Swimming Federation’ lists the annual best performance for each athlete, so results for each individual athlete were included only once in the same year. The FINA data used were for the top eight males and top eight females for each stroke and each distance per year. As FINA World championships are held every other year we were able to include five consecutive events. Ultimately, data were available for a total of 29,506 athletes, including 14,166 Swiss women and 14,798 Swiss men, and 240 women and 240 men at the international level. The study was approved by the Institutional Review Board of St. Gallen, Switzerland, and the requirement for informed consent was waived on the basis that the study used publicly available data.

To compare data for different race distances, swimming times were transformed to swimming speed (m/s). Swiss women and men were divided into ten age groups, and the three fastest swim speeds for each distance and year were determined for each group. Swimming speed of the top three athletes showed no significant difference among years (one-way ANOVA, *p* > 0.05), so swimming speeds of the top fifteen athletes were pooled for each sex/age group. From these fifteen athletes, the top three were determined for each sex, age group, and race distance. In cases where there were fewer than three swimmers, that group was excluded from the analysis. The 10–19 year and 20–29 year groups consistently showed the fastest swimming speeds, so these groups were further divided into two-year age groups, and swimming speeds of the top three athletes were determined for each sex and distance. FINA finalists were divided into two-year age groups, and swimming speeds of the top three athletes in each age group were determined for each sex and race distance. To compare sex-related difference in breaststroke and freestyle swimming, the fastest three women and fastest three men were determined for each swimming style and race distance for Swiss and FINA athletes. Sex-related differences were calculated as the absolute value of [(performance by men) – (performance by women)]/(performance by men) × 100. Sex related difference was calculated for every pairing of equally placed athletes (*e.g.* between men and women winner, between men and women 2nd place, etc*.*) before calculating mean value and standard deviation of all the pairings. In order to facilitate reading all sex differences were transformed to absolute values before analysing.

### Statistical analyses

Prior to statistical analyses, data for each sex/age/distance group were tested for normal distribution and homogeneity of variances. Normal distribution was tested using the D’Agostino and Pearson omnibus normality test [18]. Homogeneity of variances was tested using Bartlett’s test. One-way analysis of variance (ANOVA) with subsequent Tukey-Kramer post-hoc analysis was used to determine the significance of differences between gender/age/distance groups. Two-way ANOVA (age × swimming style), with subsequent Bonferroni post-hoc analysis, was used to determine the significance of the interactive effect of swimming style × age on performance. The statistical significance of differences between male and female athletes for each swimming style was determined using Student’s *t*-test. Statistical analyses were performed using IBM SPSS Statistics (Version 19, IBM SPSS, Chicago, IL, USA) and GraphPad Prism (Version 5, GraphPad Software, La Jolla, CA, USA). Significance was accepted at *p* ≤ 0.05 (two-tailed for *t*-tests). Results reported in the text and figures are means ± standard deviation (SD).

## Results

### Sex-related differences in peak swimming speed

**Figure 1:**
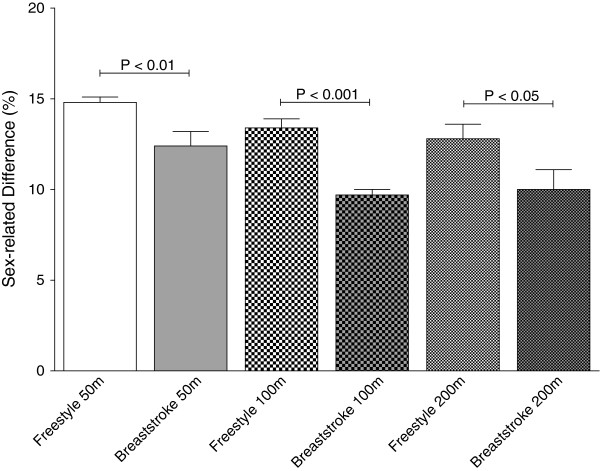
**Sex-related difference in swimming speed at national level per swimming style and distance.***P*-values indicate significant differences between sex differences of different swimming styles over the same distance. Mean ± SD.

**Figure 2:**
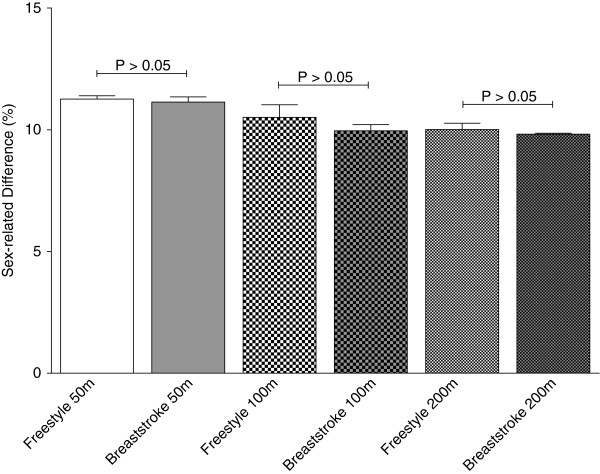
**Sex-related difference in swimming speed at international level per swimming style and distance.***P*-values indicate significant differences between sex differences of different swimming styles over the same distance. Mean ± SD.

In both Swiss and FINA results, men showed consistently faster peak swimming speeds than women (*p* < 0.01), (Table 1). The sex-related difference in peak swimming speed between the top three Swiss women and men was significantly greater for freestyle swimming than breaststroke at all distances (*p* < 0.01 for 50 m, *p* < 0.001 for 100 m, *p* < 0.05 for 200 m) (Figure 1). In the FINA results, however, sex-related differences did not differ significantly between freestyle and breaststroke (*p* > 0.05 for all distances) (Figure 2). Both Swiss and FINA data showed a significant decrease in sex-related differences for freestyle and breaststroke with increasing race distance: 12.4 ± 0.8% for 50 m vs. 10.0 ± 1.1% for 200 m in Swiss breaststroke swimmers; 11.4 ± 0.2% for 50 m vs. 9.8 ± 0.3% for 200 m in FINA breaststroke swimmers; 14.8 ± 0.3% for 50 m vs. 12.8 ± 0.8% for 200 m in Swiss freestyle swimmers; 11.3 ± 0.1% for 50 m vs. 10.0 ± 0.3% for 200 m in FINA freestyle swimmers (*p* < 0.05 in each case).

**Table 1:** Swimming speed (m/s) of the top three breaststroke and freestyle swimmers in Swiss and FINA competitions during 2006-2010

**Level**	**Top Swiss swimmers**		**FINA finalists**	
**Stroke**	**Breaststroke** **(m/s)**	**Freestyle** **(m/s)**	**Breaststroke** **(m/s)**	**Freestyle** **(m/s)**
**Men**				
50 m	1.79 ± 0.01	2.25 ± 0.03	1.87 ± 0.01	2.36 ± 0.01
100 m	1.60 ± 0.02	2.04 ± 0.02	1.71 ± 0.00	2.12 ± 0.01
200 m	1.47 ± 0.02	1.87 ± 0.02	1.57 ± 0.00	1.94 ± 0.02
**Women**				
50 m	1.55 ± 0.01	1.92 ± 0.02	1.66 ± 0.00	2.10 ± 0.01
100 m	1.34 ± 0.01	1.77 ± 0.01	1.54 ± 0.01	1.90 ± 0.02
200 m	1.33 ± 0.03	1.64 ± 0.01	1.41 ± 0.00	1.75 ± 0.02

Results are mean ± SD.

### The age of peak swimming performance

**Figure 3:**
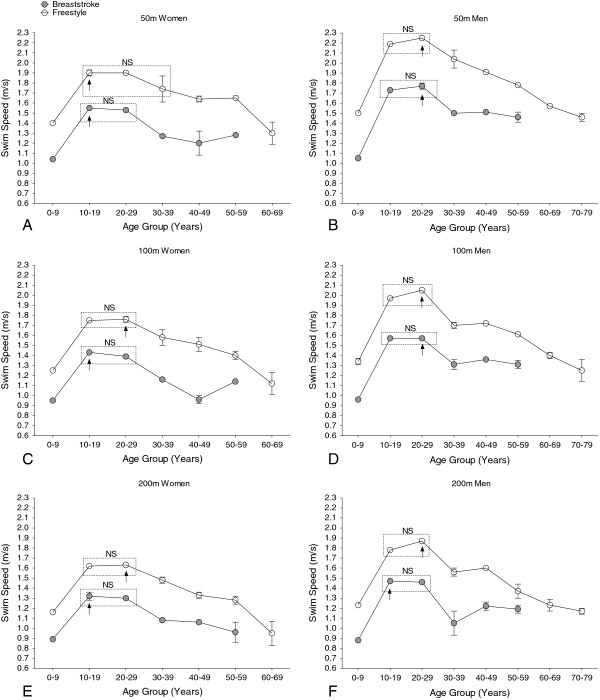
**Swimming speed at national level of women (Panels A, C, E) and men (Panels B, D, F) breaststroke (grey, filled circle) and freestyle (empty circle) swimmers between 2006 and 2010 per 10-year age group and race distance for 50 m (Panels A, B), 100 m (Panels C, D), and 200 m (Panel E, F).** Arrow denotes the age group with the numeric fastest swimming speed. Age groups with no significant difference in swimming speed are indicated by a rectangle and marked with “NS”. Mean ± SD.

The overall age of participants at national and international level was at 15.1 ± 3.4 years, whereas the mean age at national competition level was at 14.9 ± 3.2 years and at international level was at 23.3 ± 3.5 years. When Swiss swimmers were categorized into ten-year age groups, peak swimming performance by women and men in both freestyle and breaststroke swimmers for all distances consistently occurred in the 10–19 years and 20–29 years groups (Figure 3). In the 50 m freestyle, swimming speeds of women in the 30–39 years group were similar to those of the two younger groups. The interactive effect of age × swimming style on performance was highly significant (*p* < 0.001 for all distances).

**Figure 4:**
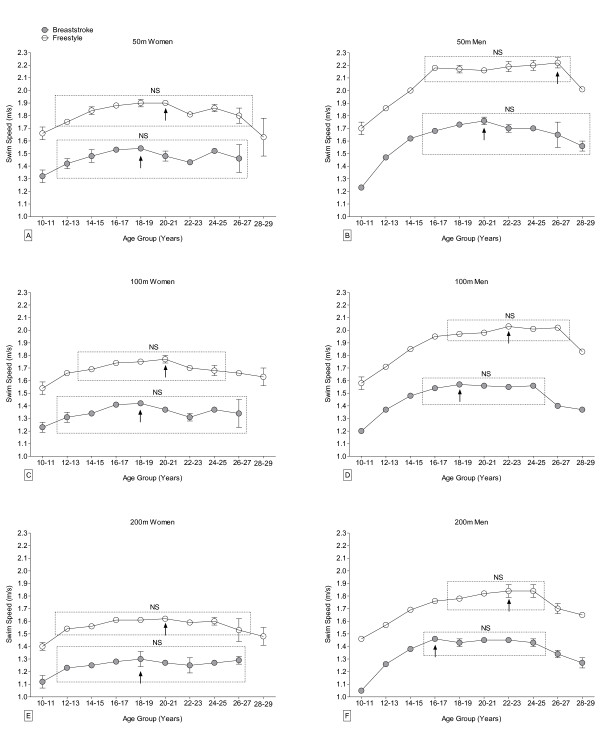
**Swimming speed at national level of women (Panels A, C, E) and men (Panels B, D, F) breaststroke (grey, filled circle) and freestyle (empty circle) swimmers between 2006 and 2010 per 2-year age group and race distance for athletes between 10 and 29 years for 50 m (Panel A, B), 100 m (Panel C, D), and 200 m (Panel E, F).** Arrow denotes the age group with the numeric fastest swimming speed. Age groups with no significant difference in swimming speed are indicated by a rectangle and marked with “NS”. Mean ± SD.

**Figure 5:**
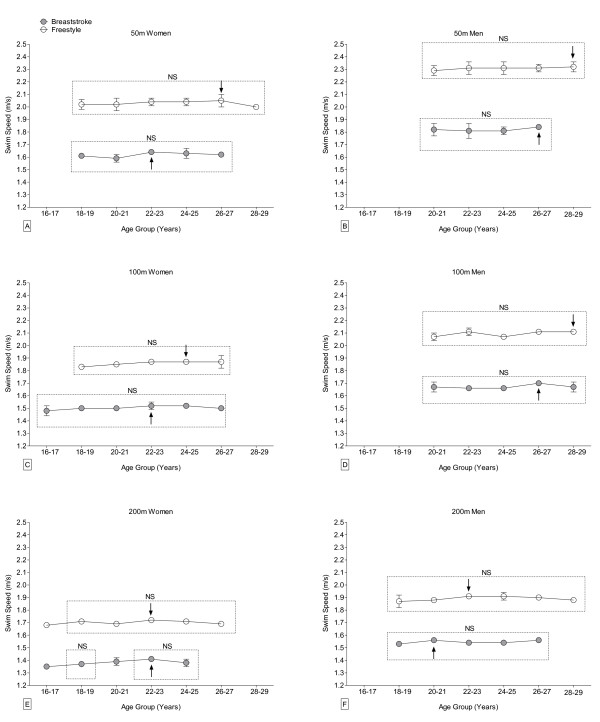
**Swimming speed at international level of women (Panels A, C, E) and men (Panels B, D, F) breaststroke (grey, filled circle) and freestyle (empty circle) swimmers between 2003 and 2011 per 2-year age group and race distance for athletes between 10 and 29 years for 50 m (Panel A, B), 100 m (Panel C, D), and 200 m (Panel E, F).** Arrow denotes the age group with the numeric fastest swimming speed. Age groups with no significant difference in swimming speed are indicated by a rectangle and marked with “NS”. Mean ± SD.

When the 10–19 years and 20–29 years groups were broken down into two-year intervals, the age of peak swimming performance was two years younger in breaststroke than in freestyle swimming for Swiss women (18.5 years vs. 20.5 years, respectively) (Figure 4; Table 2) and for FINA women (22.5 years vs. 24.5 years, respectively) (Figure 5; Table 2). Swiss men achieved peak swimming speed five years earlier in breaststroke than in freestyle swimming (18.5 years vs. 23.8 years, respectively). The difference for FINA men was only two years (24.5 years for breaststroke vs. 26.5 years for freestyle). The age of peak swimming performance was on average four years older on average for FINA women than for Swiss women irrespective of swimming style (Table 2). Peak swimming performance was achieved six years later in breaststroke and approximately three years later in freestyle for FINA men compared to Swiss men.

**Table 2:** Age of peak swimming performance of the top ten breaststroke and freestyle swimmers in Swiss and FINA competitions during the 2006-2010

**Level**	**Top Swiss swimmers**		**FINA finalists**	
**Stroke**	**Breaststroke** **(years)**	**Freestyle** **(years)**	**Breaststroke** **(years)**	**Freestyle** **(years)**
**Men**				
50 m	20-21	26-27	26-27	28-29
100 m	18-19	22-23	26-27	28-29
200 m	16-17	22-23	20-21	22-23
**Women**				
50 m	18-19	20-21	22-23	26-27
100 m	18-19	20-21	22-23	24-25
200 m	18-19	20-21	22-23	22-23

Results represent the age group with the fastest swimming speed in years.

The age of swimming peak performance in breaststroke and freestyle for Swiss and FINA men decreased significantly with increasing race distance, as did the age of peak swimming performance in freestyle for FINA women (Figures 4 and 5; Table 2). However, race distance did not significantly affect the age of peak performance in either swimming style for Swiss women, or for FINA women swimming breaststroke.

## Discussion

The main findings of the present study were, firstly, that the sex-related difference in swimming speed was significantly greater for freestyle than for breaststroke over 50 m, 100 m, and 200 m race distances for Swiss swimmers, but not for FINA finalists. Secondly, the sex-related difference for both freestyle and breaststroke swimming speeds decreased significantly with increasing race distance for Swiss and FINA athletes. Thirdly, race distance did not affect the age of peak swimming performance by women in breaststroke, but the age of peak performance was four years older for FINA women than for Swiss women. Men achieved peak swimming performance in breaststroke at younger ages for longer race distances, and the age of peak swimming performance was six years older for FINA men than for Swiss men. Fourthly, in freestyle swimming, race distance did not affect the age of peak swimming performance for Swiss women, but the age of peak swimming performance decreased with increasing race distance for Swiss men and for both sexes at the FINA World Championships.

Due to the observational and cross-sectional study design interpretation of present results is limited to some extent. Furthermore, possible influences of anthropometric, biomechanical and physiological measures could not be considered [19-21]. However, this drawback is compensated for by the large study population providing sufficient power for the statistical analyses.

### The sex-related differences in peak swimming speed

Results for elite Swiss swimmers supported the hypothesis that sex-related differences in peak swimming performance were smaller for breaststroke than for freestyle swimming. This finding can be partly attributed to the biomechanics of the swimming styles, particularly the front crawl, which is the fastest technique and typically used for freestyle swimming [20]. At high swimming speeds, women have a higher stroke rate and shorter stroke length than men, resulting in a poorer performance, and this effect is more pronounced in the front crawl than in breaststroke [22]. Furthermore, Sánchez [23] found that men have a higher stroke index (SI) than women, and that the SI is higher in freestyle than in breaststroke, which could translate into a higher swimming efficiency for women in breaststroke swimming. Indeed, Havriluk [16] found that women have a higher level of technical efficiency than men in breaststroke swimming, relative to the sex-related difference in freestyle swimming. Sex-related differences in body drag could also affect performance differences between swimming styles. Breaststroke is the swimming style with the greatest body drag, while freestyle has the least drag [24]. Female swimmers have a lower degree of body drag than men [25,26], and should have a greater gender advantage in breaststroke than in freestyle swimming. Nevertheless, women lack the absolute power to achieve comparable performance times [27] and consistently underperform men in both freestyle and breaststroke. This might be especially important in short races, where anaerobic capacity and upper extremity muscle power are most influential.

In contrast to the Swiss data, the FINA data did not support the hypothesis that sex-related differences are smaller for breaststroke than for freestyle swimming highlighting the fact that the performance difference between men and women is not solely of sex-related nature. Different skill levels at national and international level must also be taken into account. This is further supported by studies of performance determining factors, like stroke rate, arm lag time, and simultaneous arm-leg propulsion time [28]. These factors differ not only with sex but also with performance level and event. FINA competitors represent a much larger pool of elite athletes than the Swiss pool and have greater opportunities for training, particularly women. Top FINA women might be sufficiently stronger freestyle swimmers than top Swiss women to close the gender gap to some extent. In fact, the sex-related difference in freestyle swimming was smaller for FINA athletes in 50 m races than for Swiss athletes in 200 m races (10.3% vs. 12.4%, respectively). At international women have the same access to swimming training compared to men, which leads to a higher training load compared to national level. Mujika et al. [29] described a positive correlation of training load on performance in competitive swimming which might explain this circumstances [30].

Data for both Swiss and international swimmers supported the hypothesis that sex-related differences in both breaststroke and freestyle decline with increasing race distance. This result confirmed previous findings of Tanaka and Seals [12], who concluded from freestyle records that women swim more efficiently than men, and so show relative improvement in performance as race distance increases. The more economic swimming in women has been attributed to smaller body size, which reduces drag, as well as shorter legs, a greater percentage of body fat, and lower density, which results in a more horizontal and streamlined position [8,9,12].

### The age of peak swimming performance

Results of the present study did not support the hypothesis that the age of peak swimming performance is similar for breaststroke and freestyle swimming. Both Swiss and international women and men exhibited peak swimming performance at younger ages in breaststroke than in freestyle events. However, when 10-year age classes were used to analyse the data, this difference was not seen showing that the use of the finest possible age scale is important to detecting such differences.

Both Swiss and FINA data corroborated previous findings that, in freestyle swimming over distances of 50 m to 1,500 m, women generally achieve their peak swimming performance at younger ages than men [3]. Earlier maturation and puberty in women [31,32] account at least partly for this difference. Maximal increases in bone width, mineral content, and density occur earlier in women than in men [33]. The growth pattern of metacarpal bones also shows a two-year difference between sexes [31]. Lean body mass, which primarily reflects muscle mass, begins to increase during early puberty in both sexes, but females gain more fat-free mass than males [34]. Fat mass increases more during later puberty in women than in men [32], which might increase swimming efficiency, as mentioned previously.

One exception to the relatively consistent sex-related difference in the age of peak performance was in the 200 m breaststroke. Based on the age group with the numeric fastest swim, both Swiss and FINA men achieved their peak swimming speed at a younger age than women. However, the reason for that finding remains unclear and warrants further investigation. With regard to the statistical power of the underlying analysis we consider this finding rather incidental, in particular as there is no obvious physiological explanation behind.

Top female and male athletes in the FINA World Championships were 4–6 years older in breaststroke and 3–4 years older in freestyle than top Swiss swimmers. This difference can be explained by the higher performance required to successfully compete at international level. Skills and experience that take years of high training load are usually required to close the performance gap between national and international levels, and achieve qualification times for international events [30].

Finally, results of the present study showed that the age of peak swimming performance by women in breaststroke and freestyle swimming was independent of race distance in the 50 m to 200 m range. Schulz and Curnow [3] found peak swimming performance for women in 100 m freestyle events at about the same age (19.4 years) as the present study, but at a younger age (17.6 years) for 400 m races [9]. In contrast, Tanaka and Seals [12] found that women achieved their fastest swimming times for longer freestyle distances (1,500 m) at 30–35 years, and short distances (50 m) at 20–30 years [1]. However, Tanaka and Seals excluded the 10–19 years age group from their analysis, and Schulz and Curnow [3] included rather old data in theirs. The present study did not include distances greater than 200 m, so further study is needed to determine the actual age of peak performance for long freestyle and breaststroke races.

## Conclusions

The present study found greater sex-related differences in peak swimming performance in freestyle than in breaststroke for top Swiss swimmers, but not for FINA finalists. The sex-related difference decreased with increasing race distance for both swimming styles in both groups. Women consistently achieved peak swimming speed at younger ages than men. Further studies are required to better understand why the age of peak swimming performance differs between breaststroke and freestyle, and to examine sex-related differences in other swimming styles.

## Competing interests

The authors declare that they have no competing interests.

## Authors’ contributions

All authors have been involved in collecting data, writing, drafting, and revising the manuscript. MW interpreted the data, drafted, and revised the manuscript. BK conceived, designed, coordinated the study, and revised the manuscript. CAR carried out the data collection, statistical analysis, and interpretation. TR participated in the study design and revised the manuscript critically for intellectual content. RL participated in designing and coordinating the study, and revised the manuscript critically. All authors read and approved the final manuscript.

## Pre-publication history

The pre-publication history for this paper can be accessed here:

http://www.biomedcentral.com/2052-1847/5/29/prepub
